# Relationship between suicidal ideation and family problems among young callers to the Japanese crisis hotline

**DOI:** 10.1371/journal.pone.0220493

**Published:** 2019-07-30

**Authors:** Yuh Ohtaki, Shotaro Doki, Hidetoshi Kaneko, Yasuhito Hirai, Yuichi Oi, Shinichiro Sasahara, Ichiyo Matsuzaki

**Affiliations:** 1 Graduate School of Comprehensive Human Sciences, University of Tsukuba, Tsukuba, Ibaraki prefecture, Japan; 2 Faculty of Medicine, University of Tsukuba, Tsukuba, Ibaraki prefecture, Japan; 3 Soubu Hospital, Funabashi, Chiba prefecture, Japan; 4 International Institute for Integrative Sleep Medicine, University of Tsukuba, Tsukuba, Ibaraki prefecture, Japan; Chiba Daigaku, JAPAN

## Abstract

Previous studies have reported an association between family relationships and suicidal behavior, and found that people with high suicidal ideation are not likely to consult with others about their distress. An effective consulting service is therefore necessary for such individuals. Crisis hotlines are effective for reducing suicide risk, but their associated suicide ideation rate and odds ratio of family problems children remain unclear. The present study investigated the suicidal ideation rate and odds ratio of callers under 20 years of age (N = 24,333) with family problems to the Japanese crisis hotline in 2012. There were 5,242 (21.5%), 18,061 (74.2%), and 1,030 (4.2%) calls related to family problems, other problems, and both, respectively. The suicidal ideation rate and odds ratio of callers with family problems were 2.2% and 0.426, respectively. This result suggested that callers with family problems have a significantly lower rate and odds ratio for suicidal ideation compared with others. However, some associations with a high suicide ideation rate were found for individual items among callers with family problems such as abuse (20.4%), family breakdown (16.1%), and domestic violence (10.6%). Further studies are needed to understand the suicidal ideation of callers with family problems and develop more effective preventive strategies.

## Introduction

Family relationships are deteriorating in Japan; for example, there have been more than 200,000 divorces every year since 1996 [1], and the number of child abuse consultations provided in child consultation centers, which exceeded 70,000 in 2013, continues to rise [2]. Therefore, the extent to which negative family relationships affect the mental health of children is a concern.

Previous studies have reported that family relationships are associated with suicidal behavior. Freudenstein et al. [3] reported that adolescents with severe suicidal behavior tended to perceive their mothers as less caring and more overprotective compared to those with mild or no suicidal behavior. In addition, it has been reported that child maltreatment is associated with repeat presentations to the emergency department for suicide-related behaviors [4]. Saffer et al. [5] suggested that parental care might be an important risk factor for youth suicidal behavior. On the other hand, Samm et al. [6] indicated that adolescents who were satisfied with their family relationships suffered less frequently from depressive feelings and suicidal thoughts.

It has been also reported that abuse is associated with suicidal behavior. Ystgaard et al [7] reported that childhood physical and sexual abuse was significantly and independently associated with repeated suicide attempts when controlling for the effects of the other childhood adverse factors. Norman et al. [8] conducted a systematic review regarding the impact of physical abuse, emotional abuse, and neglect on long-term health, and found that the odds ratio of suicide attempts was 3.4 for physical and emotional abuse and 2.0 for neglect. Other studies have reported that about 2.5% of males and 13.5% of females have been abused in childhood, and that the attributable risk of childhood abuse on suicide attempts is about 9–20% [9, 10]. Therefore, childhood suicidal behavior is affected by family problems such as maltreatment and abuse.

Supporting people with suicide ideation and attempting to improve their mental health is therefore necessary. Shea [11] pointed out that people with suicidal ideation were not likely to consult with others about their distress. Particularly, the more serious the suicidal ideation is, the more likely they are to regard helpers as the enemy [12]. Therefore, an effective consulting service is needed for people with suicide ideation. Crisis hotlines have been shown to be effective for reducing the suicide rate among suicide attempters [13]. Other studies have also indicated that follow-up calls reduce the suicide risk of patients with a history of attempting suicide [14–16].

Rhee et al. [17] reported that in addition to reducing suicide risk, telephone therapy through consultations improved the mental status of people with suicide ideation compared with a control group. In addition, Pil et al. [18] suggested that considering its cost effectiveness, telephone consultations are an effective strategy for preventing suicide.

Doki et al. [19] reported that suicidal ideation in young people with family problems was fifth highest among 11 telephone consultation categories in Ibaraki Prefecture, Japan. Therefore, it is hypothesized that suicide ideation in young callers with family problems is also high in Japan. However, throughout Japan, the suicide ideation rate and odds ratio of young people with family problems calling the crisis hotline remain unclear. Clarifying these issues would help identify consultation items with high suicide ideation and thereby make it easier to develop appropriate support and effective suicide prevention strategies. Therefore, the present study investigated the relationship between suicidal ideation and family problems among young people calling the Japanese crisis hotline.

## Methods

### Participants

As described in the previous Japanese crisis hotline study [20], the telephone crisis hotline, Inochi No Denwa, is a well-known, volunteer-run organization with a number of branches throughout Japan. It was founded in 1971 and provides 24-hour counseling, with counselors having to complete more than 60 hours of training before taking consultations. At the time of writing, there were about 6,800 counselors in Japan. Detailed information is routinely recorded for all cases, and all data are anonymous. In the present study, data collected in 48 of the 50 call centers from January 1 to December 31, 2012, were used secondarily; data could not be collected from the remaining two call centers using the same methods due to a technological issue at the time of the study. Callers who were over 20 years of age and for whom suicidal ideation was unknown were excluded.

### Assessment items

Counselors asked callers about demographic characteristics, such as their age and sex, as well as their major problems, physical and/or mental health problems, history of suicidal behavior and self-harm, and current suicidal ideation. Counselors assessed suicidal ideation immediately after the hotline counseling based on the information collected during the session.

Major problems were classified into the following 13 categories: life events (way of life, loneliness, bereavement, accident, disaster, etc.); thoughts (view of life and death, religion, human rights, etc.); work; finances; family; spouse; school; human relationships; love; physical health problems; mental health problems; information (provision of information from call centers, gratitude toward call centers, complaints about call centers, etc.); and other (call abruptly disconnected or dropped, nuisance phone calls, etc.). Family problems comprised the following nine items: domestic violence; family breakdown; nursing; abuse; incest; child care; dissatisfaction; support; and others. These categories were established based on discussions between specialists at Inochi No Denwa. Major problem categories were listed on the data sheets given to the counselors. Counselors listened to callers and chose the most appropriate major problem category based on their own assessments. If multiple major problems were noted in the same consultation session, counselors chose up to three and then ranked them in terms of severity based on their own assessments.

### Statistical analysis

To examine the interaction of family problems and others, we categorized major problems that included only family problems as “family”, those that did not include family problems as “others”, and those that included family problems and others as “both”. To compare the suicide ideation rate between family, others, and both, suicidal ideation was categorized as “present” or “absent”. We conducted a chi-square test to investigate the relationship between suicidal ideation and each major problem.

To determine the odds ratio for suicidal ideation, binomial logistic regression was then conducted using each attribute as a covariate. We used SPSS version 24 for Windows (IBM, Tokyo) for all analyses, with 5% set as the significance level.

### Ethical considerations

This study was approved by the ethics committees of the University of Tsukuba (approval number 73) and the Inochi No Denwa Federation. The anonymity of both the counselors and the callers was preserved. Verbal informed consent was not conducted, but the Inochi No Denwa website has shown that the consultation data are anonymized and used for research.

## Results

The callers’ demographic characteristics are shown in Table 1. Data were obtained from a total of 24,333 calls. There were 5,242 (21.5%), 18,061 (74.2%), and 1,030 (4.2%) calls for the major problem categories of family, others, and both, respectively. The relationship between major problems and suicidal ideation is shown in Table 2. A significant difference was observed between the suicidal ideation rates in the categories of family, others, and both (2.2%, 7.9%, and 10.9%, respectively; chi-squared test, *p* < 0.001).

**Table 1:** Demographic characteristics of callers under 20 years of age (N = 24,333).

Attribute	N (%)
Sex	
Male	20,097 (82.6)
Female	4,236 (17.4)
Major Problem	
Family	5,242 (21.5)
Others	18,061 (74.2)
Both	1,030 (4.2)
Physical Health Problems	
None	18,679 (76.8)
Past history	362 (1.5)
Suspected	369 (1.5)
Undergoing treatment	866 (3.6)
Unknown	4,057 (16.7)
Mental Health Problems	
None	16,550 (68.0)
Past history	266 (1.1)
Suspected	1,977 (8.1)
Undergoing treatment	1,104 (4.5)
Unknown	4,436 (18.2)
History of Suicide Attempts	
No	21,249 (87.3)
Yes	532 (2.2)
Unknown	2,552 (10.5)
Suicidal Ideation	
Absent	22,681 (93.2)
Present	1,652 (6.8)

**Table 2:** Relationship between major problems and suicidal ideation.

Major Problem	SI Absent	SI Present	Chi-square	*P*
Family (%)	5,127 (97.8)	115 (2.2)	242.5	< 0.001
Others (%)	16,636 (92.1)	1,425 (7.9)		
Both (%)	918 (89.1)	112 (10.9)		

SI: Suicidal Ideation

The relationship between family problems, suicidal ideation and history of suicide attempts is shown in Table 3. Those with abuse had the highest suicidal ideation rate (20.4%), followed by family breakdown (16.1%) and domestic violence (10.6%). Those with abuse also had the highest suicide attempt rate (8.0%), followed by domestic violence (6.4%) and family breakdown (5.4%). The results of the binomial logistic regression analysis of suicidal ideation are shown in Table 4. The odds ratio for suicidal ideation was 0.426 and 1.46 for callers in the categories of family and both, respectively.

**Table 3:** Relationship between Family Problems, Suicidal Ideation and History of Suicide Attempts.

Family Problem	SI Absent (%)	SI Present (%)	HSA No (%)	HSA Yes (%)	HSA Unknown(%)
Child care	13 (92.9)	1 (7.1)	13 (92.9)	0 (0)	1 (7.1)
Support	9 (100)	0 (0)	9 (100)	0 (0)	0 (0)
Nursing	10 (100)	0 (0)	10 (100)	0 (0)	0 (0)
Family breakdown	47 (83.9)	9 (16.1)	48 (85.7)	3 (5.4)	5 (8.9)
Domestic violence	42 (89.4)	5 (10.6)	40 (85.1)	3 (6.4)	4 (8.5)
Abuse	90 (79.6)	23 (20.4)	87 (77.0)	9 (8.0)	17 (15.0)
Incest	2,607 (99.4)	17 (0.6)	2,479 (94.5)	3 (0.1)	142 (5.4)
Dissatisfaction	823 (94.4)	49 (5.6)	770 (88.3)	16 (1.8)	86 (9.9)
Others	1,486 (99.3)	11 (0.7)	1,381 (92.3)	5 (0.3)	111 (7.4)

SI: Suicidal Ideation, HSA: History of Suicide Attempts

**Table 4:** Binominal Logistic Regression of Suicidal Ideation (N = 24,333).

Attribute	B	OR	95% CI Lower	95% CI Upper	*P*
Sex					
Male (Reference)	-	-	-	-	
Female	1.24	**3.45**	**3.03**	**3.92**	**<0.001**
Major Problem					
Others (Reference)	-	-	-	-	
Family	–0.854	**0.426**	**0.342**	**0.530**	**<0.001**
Both	0.379	**1.46**	**1.12**	**1.90**	**0.005**
Physical Health Problems					
None (Reference)	-	-	-	-	
Past history	0.432	**1.54**	**1.05**	**2.26**	**0.028**
Suspected	0.578	**1.78**	**1.24**	**2.57**	**0.002**
Undergoing treatment	0.416	**1.52**	**1.19**	**1.93**	**0.001**
Unknown	0.071	1.07	0.887	1.30	0.465
Mental Health Problems					
None (Reference)	-	-	-	-	
Past history	0.576	**1.78**	**1.13**	**2.80**	**0.013**
Suspected	0.799	**2.22**	**1.83**	**2.71**	**<0.001**
Undergoing treatment	1.28	**3.59**	**2.92**	**4.43**	**<0.001**
Unknown	0.140	1.15	0.940	1.41	0.175
History of Suicide Attempt					
No (Reference)	-	-	-	-	
Yes	4.35	**77.3**	**60.1**	**99.5**	**<0.001**
Unknown	2.15	**8.61**	**7.41**	**9.99**	**<0.001**

OR; odds ratio, CI; confidence interval

Bold figures are statistically significant.

## Discussion

This study examined the relationship between suicidal ideation and family problems among young people calling the Japanese crisis hotline. Callers with family problems had a significantly lower suicidal ideation rate and odds ratio compared with others (Table 4). Higher rate of suicidal ideation and suicide attempt were seen for family problem items such as abuse (20.4%, 8.0%), family breakdown (16.1%, 5.4%), and domestic violence (10.6%, 6.4%), but there were few problems in terms of abuse (132), family breakdown (68), and domestic violence (53) (Table 3). On the other hand, lower suicidal ideation rates (incest, 0.6%; others, 0.7%; and dissatisfaction, 5.6%) were seen for items with high numbers of problems (incest, 2,624; others, 1,497; and dissatisfaction, 872). Cabinet Office [21] reported that 67.6% of callers identified their family members as counsellors for their problems; however, if their problems were associated with their family, they had difficulty with such consultations. Therefore, callers to crisis hotlines tend to have family problems, even if their problems are not associated with suicidal ideation.

Incest has been reported to be related to later suicidal ideation [22–25], but in the present study, callers with a history of incest had a low suicidal ideation rate; the reason for this could be that incest was considered a current problem. Many previous studies have reported that childhood incest is related to adolescent and adult suicidal ideation [22–25], and that it takes substantial time for incest to affect suicidal ideation. Regarding the time course, Cole and Putman [26] explained that incest prevents the formation of a self-adjustment function and can lead to suicidal behavior. Suicidal behavior is caused by the interaction between impulsivity and aggression [27], and trauma such as incest exacerbates emotional vulnerability and strengthens impulsivity and aggression, which leads to suicidal behavior [28]. Another reason for the low suicidal ideation rate among those with a history of incest is that the range of incest was not clear. Incest encompasses not only sexual behavior involving contact, but also temptation, such as when a mother regards her son as her husband [29]. Therefore, the seriousness of the situation varies. In addition, no “sexual abuse” item was included in the present study, so various sexual problems were likely to be categorized as incest. Therefore, many callers with a history of incest did not have suicidal ideation. The prevalence of incest is difficult to investigate because of cultural suppression and the accompanying sense of shame and guilt [30]. A previous study in the US reported that 1.5% of male and 8.4% of female university students had been sexually abused by a family member (266 males, 530 females) [29]. A previous study in Japan reported that 2% of healthy females had experienced some type of incest by the age of 18 years [31]. Incest is regarded as socially taboo, which makes it difficult for victims to consult with others. Therefore, it is considered that there were many callers to the crisis hotline with a history of incest who chose to remain anonymous.

The suicide ideation rate was low among callers with family problems, but high among some individual items such as family breakdown and abuse. A previous study reported that instability at home was related to suicide attempts [32], which was supported by the results of the present study. Moony et al. [33] conducted a review regarding family breakdown and reported that it was caused by the interaction between family conflict, the quality of the family relationship, maternal mental health, economic problems, frequent changes in the living environment, and family structure. Family breakdown involves dysfunction at home owing to factors such as discord, separation, and violence [34–36], and some situations are likely to cause suicidal ideation [37]. In particular, a number of previous studies have shown that physical and sexual abuse is associated with suicidal ideation [22, 38, 39]. It has also been reported that witnessing domestic violence tends to lead to suicidal ideation and behavior [40, 41]. The results of the present study support these findings. On the other hand, some factors, including qualified and considerate care, parental psychological health, little parental conflict, cooperative care after a divorce, and social support, help maintain the health of children, even in the case of a family breakdown [42, 43]. It is therefore important for counselors to examine these factors and consider using them in daily life.

## Limitations

As described in the previous Japanese crisis hotline study [20], the present study did have some limitations. First, we did not include other factors that are known to affect suicidal ideation, such as income, marital status, and social support. As low income and levels of social support tend to lead to suicidal ideation, these factors could affect its odds ratio. Second, Inochi No Denwa is not a research organization; therefore, some of the categories for major problems and suicidal ideation are ambiguous (i.e., these categories were not based on a formal questionnaire). Therefore, these categories should be revised for clarity in a future study.

As described in the Introduction, the more serious the suicidal ideation is, the more likely they are to regard helpers as the enemy [12]. It can be speculated that many people who have suicidal ideation may not have called for help. Therefore, the sample selection in this study was biased and difficult to represent the population.

Previous suicidal ideation was not investigated. However, people who have had suicidal ideation in the past are more likely to have suicidal ideation at the time of a crisis hotline call [44, 45]. Previous suicidal ideation should therefore be examined in a future study.

We did not collect additional information regarding prior suicide attempts. If counselors had asked about the frequency, number, and intent of prior suicidal attempts, this information would have enhanced the interpretation of the findings.

There are frequent callers to crisis hotlines [46], and our data may have contained duplicate callers. The data were anonymized, so duplicate data could not be excluded by comparing individual data. It is possible that the number of callers and the seriousness of suicidal ideation were overestimated. A system that can eliminate duplicate callers by asking about the number of previous consultations is therefore necessary.

Although counselors receive training, they use their own judgment to determine whether callers have suicidal ideation and what kind of consultation they should receive. Therefore, there could be bias on the part of the counselors. In addition, there were “unknown” categories in the analysis for many variables. This could distort the interpretation of the results and the assessment of suicide risk. It is therefore important to compile a manual that can help counselors identify which category callers belong to in a future study. Counselors should try to ask questions about basic attributes as naturally as possible to reduce the number of “unknown” responses.

The study design was cross-sectional and the data were anonymous; thus, it was impossible to know what happened to callers after the consultations. Longitudinal studies are needed to clarify the outcomes of callers and the effects of the consultations.

## Conclusion

This study examined the relationship between suicidal ideation and family problems among young callers to a Japanese crisis hotline. The suicidal ideation rate and odds ratio were significantly lower compared with other callers, but some associations with a high suicide ideation rate were found for individual items among callers with family problems. Further studies are needed to understand the suicidal ideation of callers with family problems and develop more effective preventive strategies.
